# Communication map of elderly people Sociodemographic and cognitive-linguistic aspects

**DOI:** 10.1590/S1980-57642013DN74000005

**Published:** 2013

**Authors:** Marcela Lima Silagi, Aline Rufo Peres, Eliane Schochat, Leticia Lessa Mansur

**Affiliations:** 1 Master in Neurology, Speech-language Pathologist of the Undergraduate Program in Speech-Language Pathology and Audiology. Department of Physical Therapy, Speech-Language Pathology and Audiology, and Occupational Therapy of the School of Medicine, Universidade de São Paulo, São Paulo SP, Brazil.; 2 Speech-Language Pathologist, Department of Physical Therapy, Speech-Language Pathology and Audiology, and Occupational Therapy of the School of Medicine, Universidade de São Paulo, São Paulo SP, Brazil.; 3 Associate professor of the Undergraduate Program in Speech-Language Pathology and Audiology. Department of Physical Therapy, Speech-Language Pathology and Audiology, and Occupational Therapy of the School of Medicine, Universidade de São Paulo, São Paulo SP, Brazil.

**Keywords:** social support, communication, language, elderly

## Abstract

Language and communication difficulties may occur in the elderly population. This is the case of the tip-of-the-tongue phenomenon and receptive and auditory comprehension difficulties. Few studies have focused on examining the effects of social exposure on maintaining communication in the aging process.

**OBJECTIVES:**

[1] To describe the communication map of healthy elderly subjects; [2] To search for associations between frequency and time dedicated to communication and cognitive and sociodemographic factors.

**METHODS:**

Healthy elderly subjects were submitted to cognitive screening, the Token Test – Revised, and the Verbal Fluency test, and answered the ASHA-FACS and the Circles of Communication Partners questionnaires.

**RESULTS:**

55 subjects, 67% female, with ages over 60 years and varied schooling were included in the sample. Interlocutors in the circle of close friends and acquaintances predominated in the communication map, although the time devoted to communication with these partners was lower than in other circles. Overall, the elderly reported no deficits in language comprehension, with some reports of the tip-of-the-tongue phenomenon. Poor performances on the Token Test – Revised and in phonemic verbal fluency along with reports of communication functionality indicated that these subjects compensate for their problems.

**CONCLUSION:**

Older subjects with lower schooling tended to predominantly communicate within the family circle. Within other circles, the number of hours devoted to communication and dialogue partners was not associated with age or schooling. The time devoted to the circle of communication with friends may indicate cognitive difficulties.

## Abstract (pt)

Dificuldades de linguagem e comunicação podem ocorrer na população idosa. Esse é o caso do fenômeno da ponta da língua e também dificuldades de recepção e compreensão auditiva. Poucos estudos se dedicam a examinar efeitos da exposição social na manutenção da comunicação, no processo do envelhecimento.

**OBJETIVOS:**

[1] Descrever o mapa de comunicação de idosos saudáveis; [2] Buscar associações entre frequência e tempo destinados à comunicação e fatores cognitivos e sociodemográficos.

**MÉTODOS:**

Foram examinados idosos saudáveis que realizaram rastreio cognitivo, Token Test-Revisado, Fluência verbal e responderam aos questionários ASHA-FAcs e Círculos de Interlocutores de Comunicação.

**RESULTADOS:**

55 sujeitos, 67% do gênero feminino, idade acima de 60 anos e escolaridades variadas compuseram a amostra. No mapa de comunicação predominaram interlocutores no círculo de amigos próximos e conhecidos embora o tempo dedicado à comunicação com esses interlocutores seja menor do que nos outros círculos. Na média geral os idosos não relataram déficits de compreensão de linguagem e houve relatos de dificuldades do tipo fenômeno de ponta de língua. Baixos desempenhos no TT-R e na fluência verbal fonêmica ao lado do relato de funcionalidade na comunicação indica que esses sujeitos compensam seus problemas.

**CONCLUSÃO:**

O número de horas de interlocução e de interlocutores não está associado à idade e escolaridade, exceto para indivíduos com maior idade e menor escolaridade os quais tendem a privilegiar a interlocução em seu círculo familiar. O tempo dedicado ao círculo de comunicação com amigos pode sinalizar dificuldades de natureza cognitiva.

## INTRODUCTION

Data from the Brazilian Institute of Geography and Statistics (IBGE) show that, in 2020, the elderly population in Brazil will number 28 million individuals, a figure set to nearly double by 2040.^1^ This signals the need to reflect on how to evaluate and propose measures to guarantee the health and functionality of these subjects.

Aging is a heterogeneous process. Individuals differ from each other over time, which reflects on how they age. Nevertheless, certain difficulties may frequently occur in the elderly population, such as failing to retrieve words during conversation (tip-of-the-tongue phenomenon),^2-4^ and receptive and auditory comprehension difficulties.^5,6^

The tip-of-the-tong phenomenon may occur in young and elderly subjects, but is more frequent in the latter group. This phenomenon refers to the failure to retrieve words, especially names and places.^3^ Affected individuals are able to retrieve the meaning, formal characteristics, such as number of graphemes, initial phoneme, syllabic length of the words, or even to recall words with similar phonological structures, but fail to access the phonological representation of certain words. In general, the lexical item can be retrieved spontaneously after some time.^5^

The association between the tip-of-the-tongue phenomenon and cognition has not yet been elucidated. Studies have investigated correlations with vocabulary, working memory,^7^ episodic memory,^8^ processing speed,^7^ and monitoring information^8^, without reaching consistent conclusions.

One of the main complaints of elderly individuals – "to hear without understanding" – arises from the decreased ability to process speech sounds, which is related to aging.^9^ Elderly also frequently complain of not understanding speech in noisy or reverberating environments. These difficulties may be associated to central auditory processing disorders (CAPD).^10^

Speech comprehension difficulties in elderly subjects is usually interpreted from a multifactorial perspective, related to peripheral and central auditory nervous system alterations and also to other deficits of a cognitive nature.^11^

Both the tip-of-the-tongue phenomenon and the auditory deficits may affect the functionality of elderly individuals. Firstly, in healthy aging, functionality is ensured by the possibility of compensating for alterations and losses and adjusting to environmental demands.^12,13^ Secondly, a stimulating environment supports neural plasticity and the functionality of elderly subjects.^14^

Although the importance of a stimulating environment is recognized, few studies have examined the effects of social exposure on maintaining communication during the aging process.^14-16^

As they grow older, elderly individuals tend to restrict their social interactions. Sensory, motor, and cognitive difficulties contribute to the limitation of interlocutors and communication situations. This restriction may favor the decrease of language activities.

Habits and life style associated to these difficulties must also be considered, such as the habit of passively watching television for long periods, or spending little time on communicating with significant interlocutors.^16^

A few assessment instruments emphasize communication as a functionality index, among them is the Clinical Dementia Rating – Expanded version (CDR-E)^17^ semi-structured questionnaire. This screening instrument presents questions regarding the elderly person's difficulties retrieving words (word finding) and receptive and auditory comprehension difficulties.

Communication mapping may also be a useful way of assessing the availability of interlocutors and the mobilization of social groups, providing the elderly individual with opportunities for interaction. The network is built from the subject's perspective, and is based on the social network theory.^18^

The individual is placed at a central point, from where concentric circles are drawn, representing the domains in which interactions take place. The interlocutors from closer circles have closer relationships with the subject. Those in farther circles are not familiar and have specific roles in communicative interactions, such as driving the car to a restaurant for lunch.

Investigating the availability of communicative situations along with language and communication complaints may contribute to the identification of available social stimuli and the elderly subject's functionality. Moreover, it may guide interventions whose aim is to promote communication.

The aims of this study were: [1] to describe the communication map of healthy elderly individuals; [2] to search for associations between frequency and time devoted to communication and cognitive and sociodemographic factors (age, gender, and schooling).

## METHODS

**Sample and methods.** The sample comprised healthy individuals, a status determined by the MOANS criteria,^19^ recruited from the community in the Western part of the city of São Paulo, through posters. The subjects were asked formally by means of a questionnaire, developed by the research team, about the following inclusion criteria: functional hearing and eyesight, absence of neurological disorders, absence of psychiatric disorders and other cognitive disorders, absence of use of psychotropic drugs, and independent lifestyle in the community. Inclusion in the study followed the consecutive order of arrival at the venue where the research was conducted, during a period of 9 months. Subsequently, the Clinical Dementia Rating – Expanded (CDR-E)^17^ questionnaire was applied to the subjects. The expanded language domain comprises questions regarding lexical retrieval and disfluencies, and receptive and auditory comprehension difficulties. On the severity scale, the absence of complaints scored 0 points; the presence of complaints regarding lexical retrieval difficulties, 0.5 points; the presence of frequent word retrieval difficulties and problems understanding complex texts, 1 point; 2 points when moderate word finding alterations that significantly interfered in communication occurred or when the subject had moderate comprehension difficulties in daily conversation; and 3 points if severe language comprehension and production were present.

Besides the CDR-E, the participants were submitted to the Mini-Mental State Examination^20,21^ and the Geriatric Depression Scale (GDS) - 15 items.^22^ Subjects with scores above 5 on the GDS and those with a CDR-E score above 0 for the domains of memory, orientation, judgment and problem solving, community relations, home and hobbies, and personal care were excluded from the sample. Subjects with a score of 0.5 on language and behavioral domains were not excluded since there is no consensus about its use as a criterion for exclusion. All selected individuals had adequate performance on the screening cognitive tests considering their schooling.

**Ethical aspects.** All subjects signed a free and informed consent form, which was previously approved by the local Research Ethics Committee (CAAE – 0034.0.198. 000-10).

**Materials and procedures.** All of the following instruments were applied to the participants.

The communication maps of the subjects were obtained using the instrument Circles of Communication Partners (CCP).^18^ The elderly subjects were asked to answer the following question: *"Who are the people you talk to?"*, and to place the interlocutors in circles: *"Write down the names and the roles they play in their relationship with you (for example, paid service providers). Put their names in the numbers of the figure below." "What is the approximate conversation time you have with each of these persons?"*

**Figure 1:**
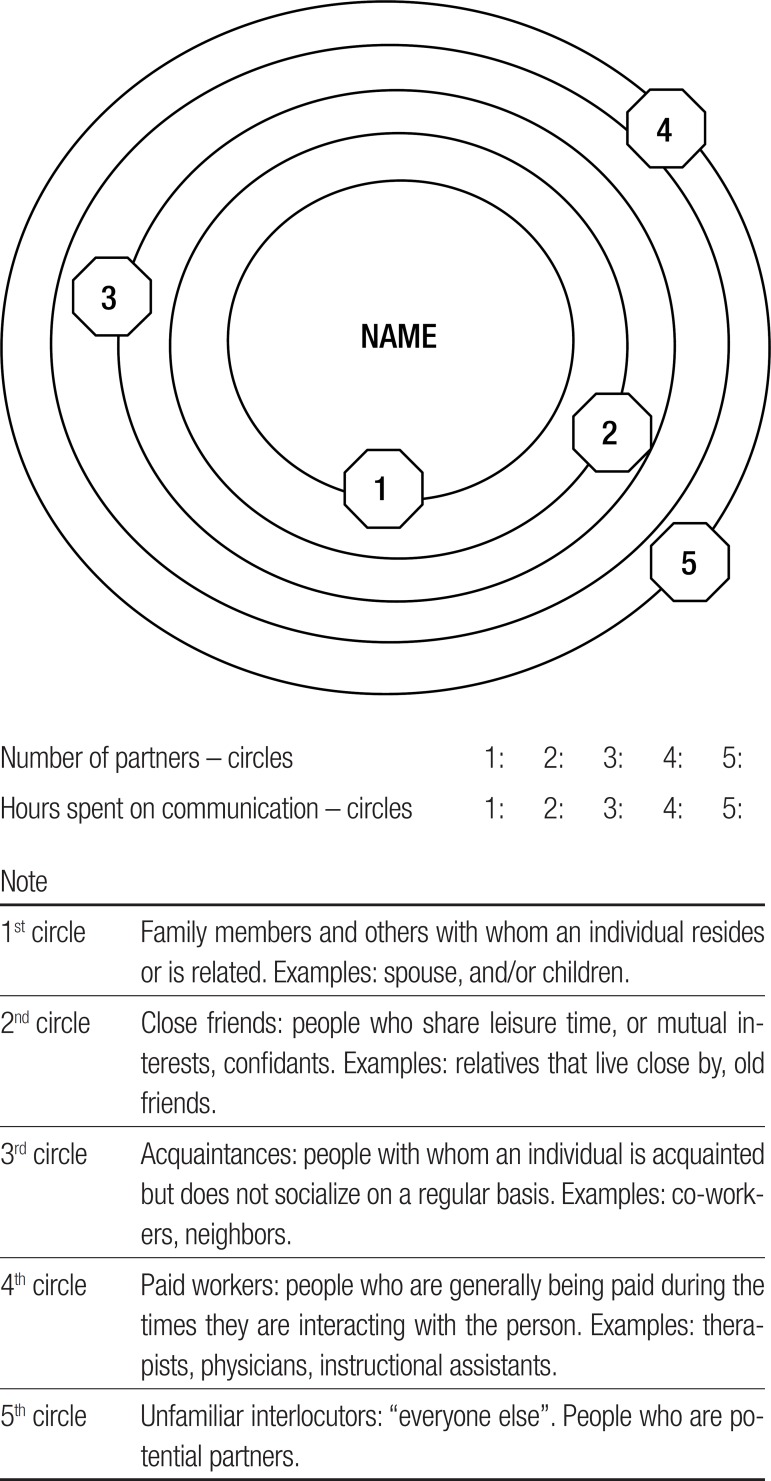
Circles of communication partners.

In addition to the CCP, the following instruments were applied:

- ASHA-Facs – Social Communication.^23^ This domain presents 21 questions regarding functionality in language comprehension and production processes. We considered independence in Social Communication, scored on a scale from 1 to 7, in which 7 represents maximum independence and 1, extreme dependence.^2^
- Phonemic verbal fluency task.^24^ The verbal fluency task according to the phonemic or literal criterion is the generation of items initiated by the letters F-A-S in a 1-minute interval, and is used to assess lexical retrieval strategies that rely on the semantic system and on executive functions. The score is given by the sum of the total numbers of items generated in one minute for each criterion, and the results were interpreted according to the Brazilian performance reference.^24^
- Revised Token Test^25^ – Subtests 9 and 10. This auditory language reception/comprehension test is also considered an instrument that assesses working memory. Twenty tokens of different shapes, sizes, and colors are used according to the examiner's instructions. In this study, the evaluation included only subtests 9 and 10, since these subtests represent the demands under study of working memory and language abilities. These have five requests each, and each request demands two actions, that in turn depend on the recognition and retention of different colors, shapes and sizes, using prepositions, prepositional phrases, adverbs, and conjunctions. There are no Brazilian standardized parameters for the Revised Token Test (RTT). Thus, as expected performance above 80% for a normal population was adopted.

**Data analyzes procedures.** The frequency of occurrence of communication partners and approximate time of interlocution were mapped in circles 1 through 5, which referred to the domains "family", "friends", "people I meet frequently, but have inconsistent sociability", "paid interlocutors", and "other distant interlocutors", as well as the time of interlocution in each circle.

In addition to the sociodemographic analysis, the statistical analysis included the description of the performances in interviews, questionnaires, tasks, and tests. Moreover, the associations (Spearman's correlations) between frequency and time of communication in the domains of the communication map with the variables gender, age, and schooling; correlations between frequency and time of communication in the domains of the communication map with depression complaints, functional communication, performance on the phonemic verbal fluency task and subtests 9 and 10 of the RTT were also analyzed. Finally, the different domains of the CCP were compared using the Wilcoxon test.

## RESULTS

In the period determined for data collection, 55 subjects were assessed, mostly females (67%), all over 60 years of age and with varied schooling. Participants participated in weekly religious or other senior groups. The sociodemographic, linguistic-cognitive, communicative performance, and language reception and production data are given in Table 1, as well as the number of communication partners in each circle and the time of interlocution.

**Table 1:** Sociodemographic, cognitive and Circles of Communication Partners (CCP) data.

	Minimum	Maximum	Mean	Standard deviation
Age (years)	60	79	68.13	5.10
Schooling (years)	0	19	8.78	5.32
MMSE (total score)	25	30	27.98	1.37
Social communication (score)	6.9	7	6.98	0.03
GDS (total score)	0	5	1.70	1.66
Phonemic verbal fluency - FAS (total number of items)	1	49	25.51	11.25
RTT - Sub-tests 9-10 correct answers (n)	1	5	2.97	0.91
RTT - Sub-tests 9-10 execution time (seconds)	37	96	49.90	9.90

RTT - Sub-tests 9-10 execution time (seconds) 37 96 49.90 9.90 MMSE: Mini-Mental State Examination; RTT: Revised Token Test; GDS: Geriatric Depression Scale.

In this sample of healthy subjects, the majority scored 0 (zero) on the CDR-E test, that is, they had no functional alterations. Seven (12.72%) individuals reported "minimal yet evident word finding alterations and disfluency in language production in daily situations", for which the score of 0.5 points was allocated in the language domain. During the interview, none of the participants reported language comprehension deficits.

The mean score of under 80% on subtests 9 and 10 of the RTT, which occurred in 48 (87.27%) participants, is noteworthy. The same pattern emerged, albeit to a lesser degree, in the phonemic verbal fluency task, on which 15 (27%) subjects had below expected performances for their age and schooling. On the other hand, the mean score in the Social Communication domain of the ASHA-Facs was adequate for all individuals. For phonemic verbal fluency, one individual had low performance, which was expected given that he was illiterate.

**Figure 2:**
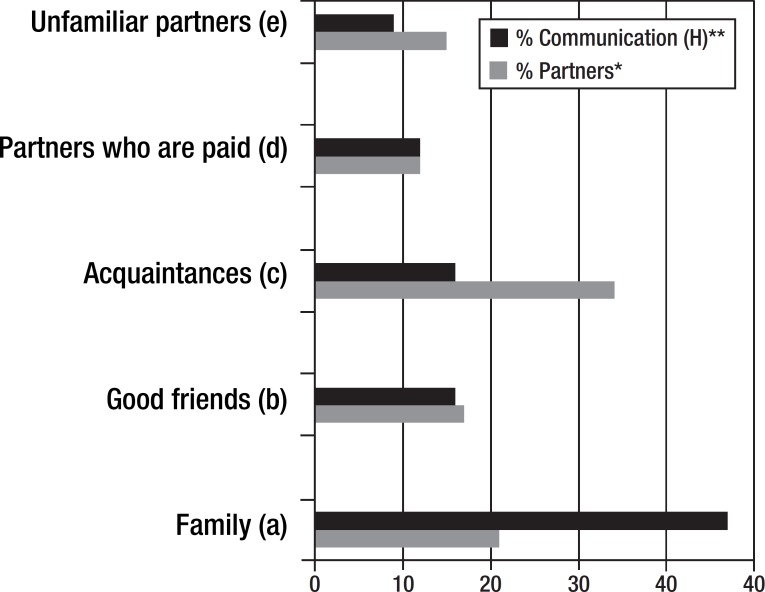
Distribution of partners and time of communication time oin the CCP*

The analysis of the five circles of the minimal communication map showed that the highest number of partners was found among "acquaintances with which there is inconsistent socialization". The number of partners in this circle was comparable with the family circle. Furthermore, the fact that the circle of "close friends" was similar to that of distant communication partners (paid partners or any potential partner) was surprising. The longest communication time occurred in the family circle, and it is noteworthy that there were no differences between the circles of close friends and acquaintances (partners with inconsistent interaction) (Figure 2).

Positive correlations, although weak, were found between the number of partners in the first circle (family) and age. Moreover, there was a negative correlation regarding the interlocutors in this circle and schooling, that is, less schooled subjects had a higher number of communication partners among family members. No correlations were found between gender and number of partners or mean time devoted to interlocution (Table 2).

**Table 2:** Spearman correlations between CCP circles, sociodemographic factors, verbal fluency, Revised Token Test, and social communication questionnaire.

			Gender	Age	Schooling	Social communication	FAS	RTT - Subtests 9-10 correct answers	RTT - Subtests 9-10 execution time
Circles of Communication Partners	1p	r	-0.13	0.325	-0.292	-0.02	0.032	-0.153	-0.182
		p	0.345	0.015	0.03	0.884	0.813	0.264	0.183
	2p	r	-0.114	0.109	0.093	0.065	0.062	0.051	0.202
		p	0.406	0.425	0.498	0.637	0.652	0.711	0.139
	3p	r	0.219	-0.059	0.059	-0.01	0.104	-0.082	0.061
		p	0.107	0.667	0.665	0.943	0.448	0.551	0.657
	4p	r	-0.167	-0.073	-0.151	-0.172	-0.11	-0.018	0.066
		p	0.222	0.598	0.27	0.209	0.424	0.895	0.633
	5p	r	-0.034	-0.041	0.096	0.074	-0.14	-0.039	-0.023
		p	0.808	0.764	0.485	0.59	0.304	0.778	0.869
	1h	r	-0.172	0.110	0.093	0.065	0.062	-0.005	-0.084
		p	0.209	0.425	0.498	0.637	0.652	0.973	0.540
	2h	r	-0.029	-0.073	-0.151	-0.172	-0.110	0.022	-0.341
		p	0.833	0.598	0.270	0.209	0.424	0.872	0.011
	3h	r	0.043	0.230	-0.265	0.053	-0.029	-0.099	-0.164
		p	0.757	0.092	0.051	0.701	0.833	0.474	0.231
	4h	r	0.024	0.010	0.081	-0.029	0.088	0.056	0.135
		p	0.860	0.945	0.555	0.836	0.521	0.686	0.326
	5h	r	-0.006	0.022	0.063	0.062	-0.157	-0.106	0.034
		p	0.965	0.876	0.647	0.653	0.251	0.442	0.807

1, 2, 3, 4, 5p: Partners in the communication domains; 1, 2, 3, 4, 5h: Hours or interlocution/week; RTT: Revised Token Test; CDR: Clinical Dementia Rating; FAS: Phonemic Verbal Fluency Task

There were no correlations between the communication map and the phonemic verbal fluency task (FAS), however, negative correlations were found between performance time in sets 9 and 10 of the RTT and time devoted to communication with close friends.

## DISCUSSION

In this sample, there was a predominance of elderly individuals younger than 75 years old. Significant language and communication deficits are not expected at this age.^16^ Nevertheless, subjects had low performance on the RTT and on the verbal fluency task (FAS) while the Social Communication domain of the ASHA-Facs questionnaire showed that they were independent and adequate in this aspect. These data suggest that elderly individuals may present problems in the sets that demand working memory abilities in the RTT and with executive demands in the FAS. These difficulties, however, are compensated in functional situations of social interaction, as observed in the ASHA-Facs – Social Communication questionnaire.

There is no prevalence data for the tip-of-the-tong phenomenon in the Brazilian population. A recent study conducted with 106 participants, from a U.S. community, observed that the complaint of tip-of-the-tongue difficulties was one of the most prevalent in a questionnaire regarding functionality and memory in daily life.^4^ Obtaining data on the occurrence of this phenomenon in our population is needed for further discussion of its cognitive interfaces.

The apparent discrepancy of answers about language functionality provided in the ASHA-Facs – Social Communication questionnaire and in the CDR-E warrants emphasis. The ASHA-Facs questions cover abilities and, the CDR-E, problems and difficulties. It is possible that directly questioning about problems encourages the perception and recognition of these difficulties. On the other hand, these difficulties are mild and do not hinder the exercise of social communication, constituting a subjective complaint. Hearing difficulties were possibly not acknowledged, given their discrete impact on the functionality of communication.

In the communication map, the older subjects were closer to their families, which is unsurprising, except for the fact that these individuals are still active and independent. Furthermore, the less schooled participants also tended to favor interlocution with their family members. Although the existence of a correlation between schooling and the network of communication partners is not surprising, we are unaware of studies that have analyzed this association. This result warrants further investigation.

Another interesting finding was the similar results regarding the variables number of partners and time devoted to communication between close friends and partners with inconsistent socialization, paid communication partners, and others. The literature on social inclusion, functionality and cognition of the elderly places value not only on the number of partners, but also the quality of interlocution, acknowledging that the affective closeness of a "confident" is just as important as the availability of a communication partner.^26,27^ From a cognitive point of view, longer communications are more indicative of the attempt to "maintain and deepen conversation topics" and of attentional, executive and semantic demands. Hence, it is interesting to note that the quality of communication is not only expressed in the number of partners, and that our subjects could explore the potential of available cognitive stimulation in social situations, increasing the time devoted to communication with close partners.

In this study, the tip-of-the-tongue phenomenon did not hinder the functionality of communication. However, it is not possible to determine the extent of its impact on the motivation to search for communication situations.

We also observed a negative association between the time taken on the RTT sets that recruit working memory and the time devoted to communication with close friends, as found in the CCP. The analysis of the time devoted to friends cannot be carried out from a unilateral point of view, and it would be unwise to assume the impact of working memory on this social habit. This interesting finding should be confirmed through further cognitive investigations. It is noteworthy that auditory comprehension difficulties were not reported by the subjects. Thus, it is possible that the time of communication with significant partners may be a direct index of difficulties.

We did not find correlations between the variables number of communication partners and time devoted to communication in the CCP and subjects' performance on the verbal fluency task, which is a measure of executive function. Our literature search retrieved no studies that have searched for this association.

The main limitation of this study was the fact that the CDR-E was applied to the subjects themselves. On the other hand, this procedure allowed observation of their subjective perception of difficulties. The sample size was also a limiting factor to be overcome in further investigations.

The main contributions of this study were to verify correlations between communication habits (partners and time of communication) and sociodemographic aspects, as well as to observe possible correlations between communication habits and cognitive-linguistic aspects.

The potential of the communication mapping method in guiding actions to maintain functionality should be recognized.^28^ Although the network of social partners does not imply, by itself, social inclusion, it may serve as a screening method for the identification of potential physical or psychological barriers related to the individual and the environment. Such hindrances constitute risk factors for the elderly subject's social isolation.

In conclusion, describing the communication map of healthy elderly subjects allowed the characterization of their usual communication partners and time devoted to communication. Frequent difficulties found in this group of subjects, such as language comprehension and lexical retrieval, seem to be compensated in functional situations.

Except for the correlations between older individuals and lower schooling favoring interlocution within their family circle, the hours of communication and number of partners were not correlated to age and schooling in the other circles. The time devoted to the circle of communication with friends may suggest cognitive difficulties.

The detection of restrictions in situations of communication may guide interventions with the aim of optimizing the environment and stimulating functionality.
